# Institutional Variability in Representation of Women and Racial and Ethnic Minority Groups Among Medical School Faculty

**DOI:** 10.1001/jamanetworkopen.2022.47640

**Published:** 2022-12-20

**Authors:** Alexander Yoo, Peggy Auinger, Jane Tolbert, David Paul, Jeffrey M. Lyness, Benjamin P. George

**Affiliations:** 1 Division of Sleep Medicine, Center for Sleep and Circadian Neurobiology, Department of Medicine, University of Pennsylvania Perelman School of Medicine, Philadelphia; 2 Department of Neurology, University of Rochester Medical Center, Rochester, New York; 3 University of Rochester Center for Health and Technology, Rochester, New York; 4 Office of Academic Affairs, School of Medicine & Dentistry, University of Rochester, Rochester, New York; 5 Department of Neurosurgery, University of Rochester Medical Center, Rochester, New York; 6 Department of Psychiatry, University of Rochester Medical Center, Rochester, New York

## Abstract

**Importance:**

Bolstering the ranks of women and underrepresented groups in medicine (URM) among medical faculty can help address ongoing health care disparities and therefore constitutes a critical public health need. There are increasing proportions of URM faculty, but comparisons of these changes with shifts in regional populations are lacking.

**Objective:**

To quantify the representation of women and URM and assess changes and variability in representation by individual US medical schools.

**Design, Setting, and Participants:**

This retrospective cross-sectional study assessed US medical school faculty rosters for women and URM, including American Indian and Alaska Native, Black, Hispanic, and Native Hawaiian or other Pacific Islander faculty. US allopathic medical schools participating in the Association of American Medical Colleges (AAMC) Faculty Administrative Management Online User System from 1990 to 2019 (updated December 31 for each year), were included. Faculty data were analyzed from yearly cross-sections updated as of December 31 for each year from 1990 to 2019. For census data, decennial census data were used for years 1990, 2000, and 2010. Intercensal estimates were used for all other years from 1990 to 2019.

**Main Outcomes and Measures:**

Trends and variability in representation quotient (RQ), defined as representation of a group within an institution’s faculty compared to its respective US county.

**Results:**

There were 121 AAMC member institutions (72 076 faculty) in 1990, which increased to 144 institutions (184 577 faculty) in 2019. The median RQ of women faculty increased from 0.42 (IQR, 0.37-0.46) to 0.80 (IQR, 0.74-0.89) (slope, +1.4% per year; *P* < .001). The median RQ of Black faculty increased from 0.10 (IQR, 0.06-0.22) to 0.22 (IQR, 0.14-0.41) (slope, +0.5% per year; *P* < .001), but remained low. In contrast, the median RQ of Hispanic faculty decreased from 0.44 (IQR, 0.19-1.22) to 0.34 (IQR, 0.23-0.62) (slope, −1.7% per year; *P* < .001) between 1990 and 2019. Absolute total change in RQ of URM showed an increase; however, the 30-year slope did not differ from zero (+0.1% per year; *P* = .052). Although RQ of women faculty increased for most institutions (127 [88.2%]), large variability in URM faculty trends were observed (57 institutions [39.6%] with increased RQ and 10 institutions [6.9%] with decreased RQ). Nearly one-quarter of institutions shifted from the top to bottom 50th percentile institutional ranking by URM RQ with county vs national comparisons.

**Conclusions and Relevance:**

The findings of this cross-sectional study suggest that representation of women in academic medicine improved with time, while URM overall experienced only modest increases with wide variability across institutions. Among URM, the Hispanic population has lost representational ground. County-based population comparisons provide new insights into institutional variation in representation among medical school faculty.

## Key Points

**Question:**

Compared with county demographic data, has representation among women and underrepresented racial and ethnic minority groups in medicine (URM) within medical school faculty changed over time, and does it vary across institutions?

**Findings:**

In this cross-sectional study of faculty from 144 US medical schools from 1990 to 2019, the median representation quotient for women increased from 0.42 to 0.80. The median representation quotient for URM increased from 0.16 to 0.24, but the trend was not significant, and variability across institutions was high.

**Meaning:**

These findings suggest that representation of women in academic medicine increased over time, whereas URM experienced only modest increases in representeation with wide variability across institutions.

## Teaser

This cross-sectional study examines institution-level trends and variability in representation of women and racial and ethnic minority groups underrepresented in medicine among academic faculty in comparison with regional demographics.

## Introduction

Health sciences inequities by gender, race, and ethnicity remain a significant problem in the US,^1,2,3,4^ whereas population diversity continues to grow.^5^ Increasing diversity within the physician and health scientist workforce is a critical issue because it directly impacts health care access, quality, and research for women and underrepresented groups.^6^ Medical schools hold special importance in addressing this gap given their responsibility in selecting and training future physicians and researchers, their ties to large regional health care institutions, and their role in supporting and cultivating research aimed at solving inequities. Therefore, there has been interest in assessing trends in faculty diversity.

Prior studies evaluated changes in representation by examining the proportion of women and underrepresented groups in medicine (URM) and found varying results across clinical departments,^7,8,9,10,11,12^ but support a collective increase.^12,13,14^ However, scrutiny of changes against a population benchmark is provided in only a handful of instances,^15,16^ with results suggesting worsening representation of URM compared with national trends. While these reports provide important context, use of national statistics assumes identical institutional objectives and discounts the impact of regional demographics on the ability of institutions to recruit and retain faculty from underrepresented groups.^17^

The primary objective of our analysis was to examine institution-level trends and variability in representation of women and URM among academic faculty in comparison to regional demographics. We hypothesized that despite increasing diversity among faculty, comparison with county population demographic data would result in less optimistic trends. We further hypothesized that between-institution variability is high and that county-level comparisons may reveal more severe gaps in URM representation than suggested by national statistics. Insights from this analysis may generate policies that will shape medical school faculty to better reflect regional populations. We therefore assessed proportions of sex and race and ethnicity among medical school faculty against matched county-level data using Association of American Medical Colleges (AAMC) and US Census Bureau data from 1990 to 2019 to uniquely contextualize changes in faculty representation.

## Methods

We used AAMC FAMOUS (Faculty Administrative Management Online User System) data on sex, race, and ethnicity for full-time medical school faculty aggregated at the institution-level linked to US Census Bureau county demographics from 1990 to 2019 (updated December 31 for each year), to assess representation of women and URM among academic faculty. Both databases contain information based on self-identification. We defined URM as American Indian or Alaska Native, Black, Hispanic, or Native Hawaiian or other Pacific Islander, consistent with prior studies (eMethods in Supplement 1).^8,12,13,16^ Our study was exempt from informed consent by the University of Rochester Research Subjects Review Board due to lack of individual identifiers. Our study followed the Strengthening the Reporting of Observational Studies in Epidemiology (STROBE) reporting guideline.

### Representation Quotient

The main outcome measure of our study was the representation quotient (RQ),^18^ calculated for individual institutions. The RQ was defined as a measure of faculty representation of a specific group (ie, proportion of total faculty) compared with the group’s representation within the matched (ie, occupied) US county. We obtained RQs by dividing the proportion of a faculty subgroup by the proportion of that subgroup in the matched US county. An RQ of less than 1.00 suggests underrepresentation of a subgroup within faculty compared with the US county population. The RQs for individual institutions were shown in distributions over time or by institutional ranking. In sensitivity analysis, RQs derived from national averages were calculated and compared with RQs calculated from matched US county-level populations.

### Inclusion and Exclusion Criteria

We collected institution-level characteristics using publicly available sources (eMethods in Supplement 1).^19^ Allopathic US medical schools with at least 3 consecutive years of data in the AAMC FAMOUS database were included (eMethods in Supplement 1). There were 144 institutions and 3 729 888 faculty entries included over the time frame (121 institutions with complete data for the 30-year time frame) (eFigure 1 in Supplement 1).

### Statistical Analysis

Faculty data were analyzed from yearly cross-sections updated as of December 31 for each year from 1990 to 2019. For census data, decennial census data were used for the years 1990, 2000, and 2010. Intercensal estimates were used for all other years from 1990 to 2019.The proportions of total faculty and county-based RQ by subgroup were calculated by each individual institution, and the distribution was characterized descriptively. Trends were assessed using every year from 1990 (or first year of data entry) through 2019, with a mixed-model repeated-measures approach to estimate the mean change per year (linear slope) for each institution. An autoregressive correlation (AR[1]) covariance structure was assumed to model the within-institution variability. Linear slopes were compared with zero using a significance level of *P* = .05. No adjustments for multiple comparisons were made. Univariate comparisons of RQ across institution variables were performed using Wilcoxon rank sum or Kruskal-Wallis tests. Comparisons between county-based and national-based RQs were demonstrated using a parallel-coordinates graph by institutional ranking in 2019. A 2-sided *P* value of less than or equal to .05 was considered statistically significant. Analyses were performed using SAS, version 9.4 (SAS Institute Inc).

## Results

### Trends in County-Based Representation Quotient

**Figure 1.**
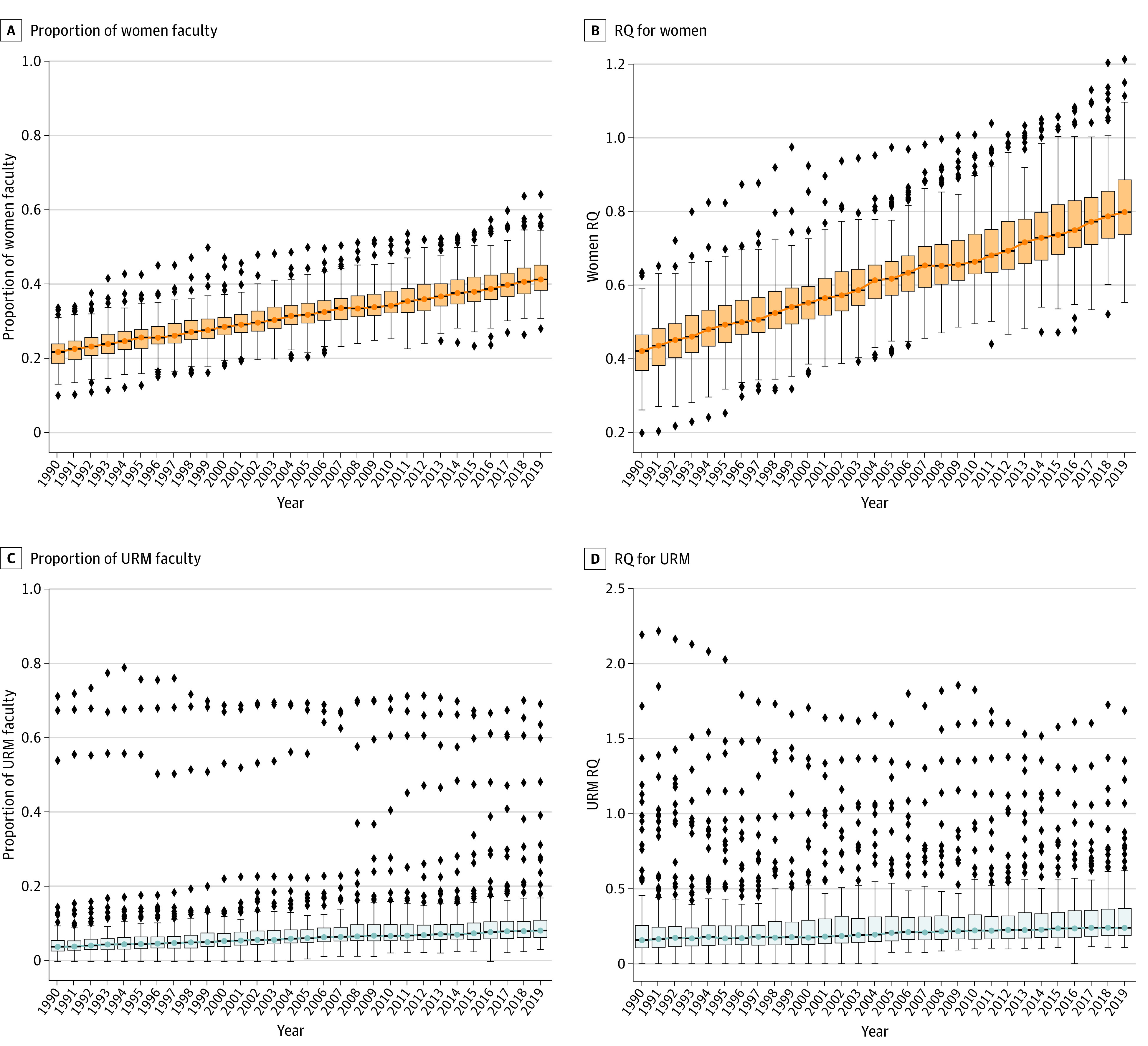
Trends and Distribution of the Proportion and Representation Quotient (RQ) for Women and Underrepresented Groups in Medicine (URM) Among Medical School Faculty, 1990 to 2019 The distribution of individual institutions is displayed using box and whisker plots. The line within each box represents the median, the outer lines of each box represent the first and third quartiles, the whiskers represent 1.5 times the value of the IQR from the first and third quartiles, and diamonds represent outliers. A and C, Proportion of faculty within an institution who identified as women and URM, respectively. B and D, RQ in which the proportion of faculty who identified as women and URM, respectively, is divided by the respective proportion of the subgroup in the institution’s US county population.

There were 121 member institutions with 72 076 total faculty (3648 [5.1%] URM faculty) in 1990 and 144 member institutions with 184 577 total faculty (17 029 [9.2%] URM faculty) in 2019 (eFigure 2 in Supplement 1). Comparison of faculty with matched county demographics revealed increases in the median RQ for women faculty from 0.42 (IQR, 0.37-0.46) to 0.80 (IQR, 0.74-0.89), a 90% relative increase from 1990 to 2019 (slope, +1.4% per year; *P* < .001) (Figure 1). Representation of URM faculty by county-based RQ increased from 1990 to 2019, but the trend was not significant (median RQ, 0.16 [IQR, 0.11-0.26] to 0.24 [IQR, 0.19-0.37], a 50% relative increase; slope, +0.1% per year; *P* = .052) (Figure 1). Among individual URM-designated races and ethnicities, RQ for Black faculty experienced a large relative, but modest absolute, increase from 1990 to 2019 (median RQ, 0.10 [IQR, 0.06-0.22] to 0.22 [IQR, 0.14-0.41], a 108% relative increase; slope, +0.5% per year; *P* < .001) (Table). There was a 22% relative decrease in RQ for Hispanic faculty over the 30-year period (median RQ, 0.44 [IQR, 0.19-1.22] to 0.34 [IQR, 0.23-0.62]; slope, −1.7% per year; *P* < .001).

**Table.** Medical School Faculty Representation of Women, Underrepresented Groups in Medicine, and Race and Ethnicity Subgroups, 1990 to 2019

Group or subgroup	Proportion of total faculty, median (IQR)^a^		Slope estimate^c^	*P* value^d^	RQ, median (IQR)^a^^,^^b^		Slope estimate^c^	*P* value^d^
	1990	2019			1990	2019		
Women	0.22 (0.19-0.24)	0.41 (0.38-0.45)	0.007	<.001	0.42 (0.37-0.46)	0.80 (0.74-0.89)	0.014	<.001
Underrepresented in medicine	0.04 (0.03-0.06)	0.08 (0.06-0.11)	0.002	<.001	0.16 (0.11-0.26)	0.24 (0.19-0.37)	0.001	.052
Race and ethnicity subgroup								
American Indian or Alaska Native	0 (0-0.002)	0.001 (0-0.002)	<0.001	.04	0 (0-0.56)	0.32 (0-0.87)	0.006	.26
Asian	0.07 (0.05-0.09)	0.20 (0.16-0.25)	0.004	<.001	3.52 (1.70-6.14)	4.01 (2.41-6.13)	0.016	.03
Black	0.02 (0.01-0.03)	0.04 (0.02-0.05)	0.001	<.001	0.10 (0.06-0.22)	0.22 (0.14-0.41)	0.005	<.001
Hispanic	0.02 (0.01-0.03)	0.04 (0.03-0.06)	0.001	<.001	0.44 (0.19-1.22)	0.34 (0.23-0.62)	−0.017	<.001
Native Hawaiian or other Pacific Islander^e^	0 (0-0)	0 (0-0.001)	<0.001	.051	NA	0 (0-1.99)	0.016	.80
White	0.86 (0.81-0.89)	0.64 (0.57-0.70)	−0.006	<.001	1.23 (1.04-1.55)	1.22 (0.98-1.54)	0.001	.33

Abbreviations: NA, not available; RQ, representation quotient.

^a^ If the median value was zero or the IQR included zero, then a value of zero is shown.

^b^ Calculated by dividing the proportion of a faculty subgroup within an institution by the proportion of that subgroup in the matched US county. Representation quotients of less than 1.00 suggest underrepresentation of a subgroup within medical school faculty ^c^Indicates the change per year as calculated by linear mixed-effects models with a maximum likelihood approach and an autoregressive correlation structure with year (ie, 1990-2019) as the dependent variable and institution as the repeated measure.

^d^ Calculated with the *t* test by comparing the linear slope to zero.

compared with the US county population. There were no faculty identifying with Native Hawaiian or other Pacific Islander for any institutions in 1990 and therefore NA is displayed.

^e^ The US Census Bureau did not identify Native Hawaiian or other Pacific Islander individuals until 2000; therefore, the RQ in 1990 was not available for this subgroup and the slope estimate reflects trends from 2000 to 2019.

### Institutional Variability

**Figure 2.**
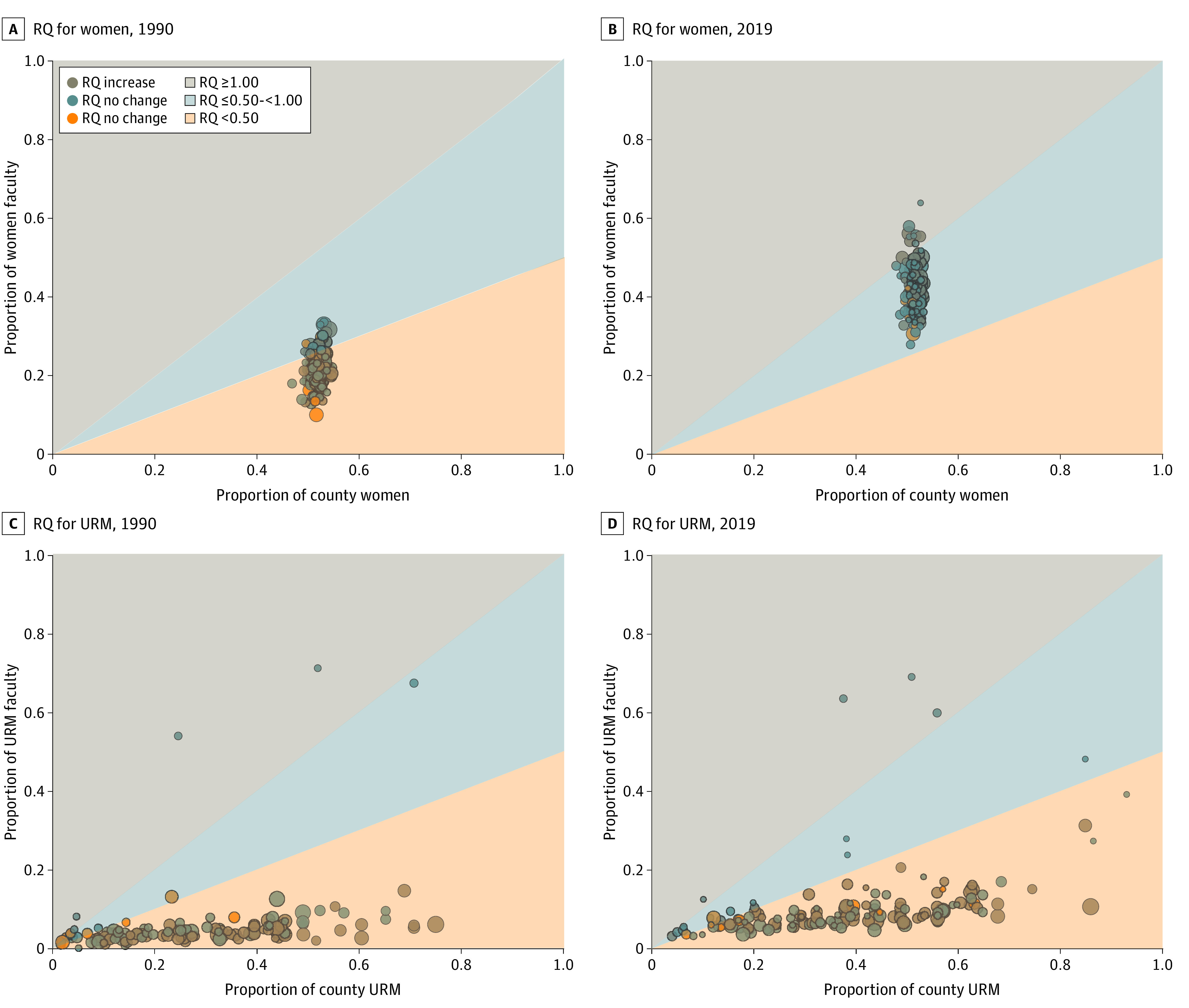
Proportion of Medical School Faculty by Proportion of County Representation for Women and Underrepresented Groups in Medicine (URM), 1990 and 2019 Each bubble represents an individual institution. Bubble size is based on faculty size. The color of each bubble demonstrates change from 1990 to 2019. The shaded area represents the county-based representation quotient (RQ).

Large variability was observed across institutions in the proportion of women and URM faculty (Figure 1 and Figure 2). The institutional distribution for proportion of faculty widened over time for women (IQR, 18.7%-23.9% in 1990 to 38.3%-45.0% in 2019) and URM (IQR, 2.8%-5.6% in 1990 to 6.4%-11.1% in 2019) (Table and Figures 1 and 2). Adjustment for county-level demographics using the RQ resulted in greater variability in the institutional distribution of representation among women (IQR, 0.37-0.46 in 1990 to 0.74-0.89 in 2019) and URM (IQR, 0.11-0.26 in 1990 to 0.19-0.37 in 2019) compared with that seen for the proportion of faculty (Figure 1).

In 1990, 18 of 121 institutions (14.9%) exceeded an RQ for women of 0.50, whereas all institutions exceeded this level by 2019, but only 11 of 144 institutions (7.6%) had RQ of 1.00 or greater for women faculty (Figure 2). Four institutions experienced an absolute decrease in RQ for women faculty over the time frame (eFigure 3 in Supplement 1). The median absolute change in RQ for women in institutions from the earliest year of data to 2019 was +0.37 (IQR, +0.29 to +0.43) (eFigure 3 in Supplement 1). There were 127 institutions (88.2%) that demonstrated a positive slope with 95% CIs above zero in RQ for women faculty from 1990 to 2019; 17 institutions had CIs that crossed zero (eFigure 3 in Supplement 1).

In 1990, 16 of 121 institutions (13.2%) exceeded RQ for URM of 0.50; 19 of 144 (13.2%) exceeded this level in 2019 (Figure 2). In 1990, there were 6 institutions (5.0%) with an RQ for URM faculty of 1 or greater, which declined to 4 institutions (2.8%) in 2019 (Figure 2). There were 34 institutions (23.6%) that experienced an absolute decline in RQ for URM faculty (eFigure 3 in Supplement 1). The median absolute change in RQ from earliest year of data to 2019 was +0.06 (IQR, +0.01 to +0.12) (eFigure 3 in Supplement 1). There were 57 institutions (39.6%) that demonstrated a positive slope with 95% CIs above zero in RQ for URM faculty from earliest year of data to 2019, and 10 institutions (6.9%) demonstrated a negative slope with 95% CIs below zero in RQ for URM faculty (eFigure 3 in Supplement 1); 77 institutions had CIs that crossed zero. Variability in county-based RQ for URM appeared to be heavily driven by differences across counties in respect to the proportion of URM individuals (eFigure 4 and eTable 1 in Supplement 1). Institutions in counties with high URM representation tended to have lower RQs.

### Contrasting National and County-Based Representation Quotients

**Figure 3.**
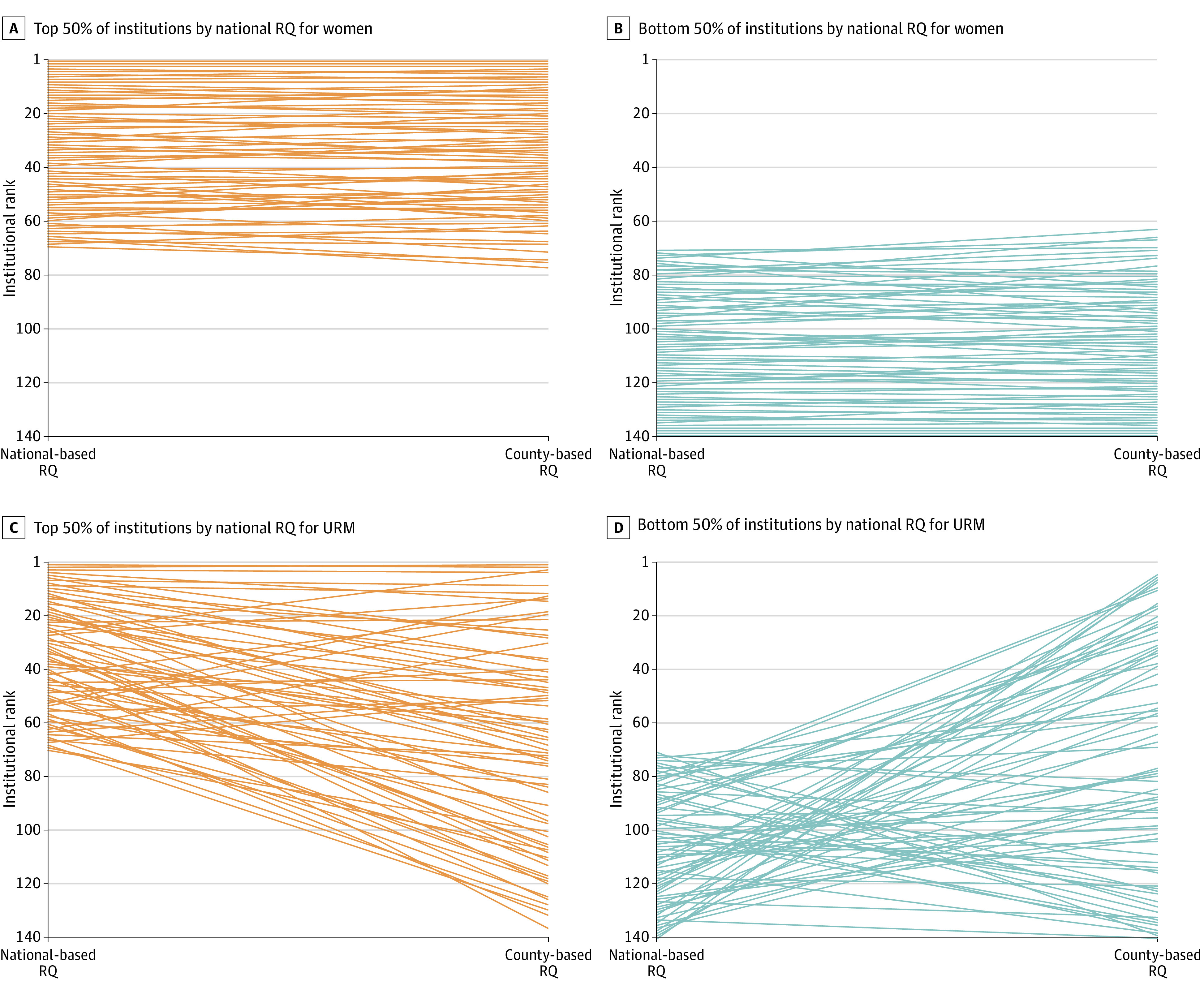
Disaggregated Parallel Coordinates Plot of County and National Representation Quotient (RQ) by Institutional Ranking for Women and Underrepresented Groups in Medicine (URM) Among Medical School Faculty, 2019 Institutional ranking by national- and county-based RQ in 2019 is displayed in a parallel-coordinates plots for women (A and B) and URM (C and D) to show institutional ranking variability between the 2 metrics. Data are displayed in rank order with the highest ranked institution having the highest RQ for the respective population. Institutions with high national-based RQ in 2019 are shown in orange (A and C) and institutions with low national-based RQ in 2019 are shown in blue (B and D).

Counties varied considerably in respect to proportion of URM. Comparison with matched national demographics resulted in vastly different institutional ranking by RQ for URM. Figure 3 demonstrates the changes in institutional ranking by RQ using a parallel-coordinate plot among the 144 institutions with complete data in 2019. For URM RQ in 2019, 33 institutions (22.9%) crossed from the top 50th percentile to the bottom 50th percentile when shifting from a national to a county-based comparison. Of these institutions, 27 were resided in urban counties for which the median percent of URM individuals was 55.9% (IQR 46.7%-60.1%) which is greater than the median for all counties with medical schools in this analysis 38.2 (IQR 23.8%-52.3%) and the national average (31.9%) in 2019.

### Institutional Characteristics

Institutional characteristics associated with high county-based RQ for women in 2019 included urban campus setting (0.82 [IQR, 0.75-0.90]; *P* = .04), West US region (0.88 [IQR, 0.81-0.98]; *P* = .005), and high RQ for women in 1990 (0.85 [IQR, 0.74-0.96]; *P* = .01) (eTables 1 and 2 in Supplement 1). Institutional characteristics associated with high county-based RQ for URM in 2019 included public medical school ownership (0.28 [IQR, 0.21-0.42]; *P* < .001), suburban (0.29 [IQR, 0.21-0.42]) and rural (0.77 [IQR, 0.39-0.88]) campus settings (*P* < .001), historically Black college and university status (1.35 [IQR, 1.07-1.69]; *P* = .003), later year established (ie, 1960-2017) (0.29 [IQR, 0.21-0.39]; *P* = .004), first quartile (lowest) faculty size (0.34 [IQR, 0.22-0.60]; *P* = .002), high RQ for URM in 1990 (0.45 [IQR, 0.37-0.68]; *P* < .001), and first quartile (lowest) of the county proportion of URM (0.43 [IQR, 0.37-0.55]; P < .001) (eTables 1 and 2 in Supplement 1). Institutional characteristics associated with low county-based RQ for URM included better institutional ranking in *US News & World Report* (0.27 [IQR, 0.20-0.39]; *P* = .02) (eTable 1 in Supplement 1).

### Faculty Rank

**Figure 4.**
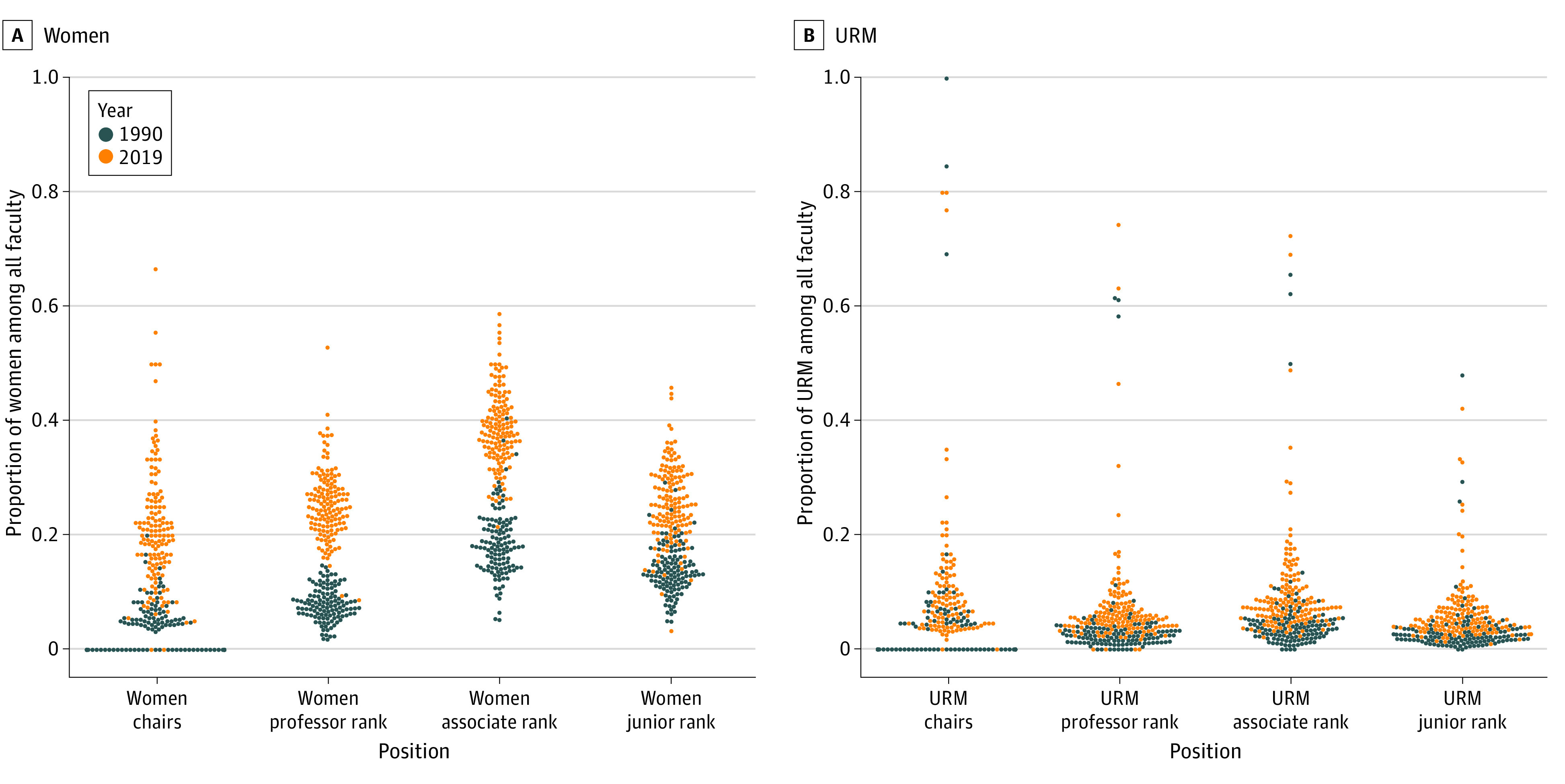
Institution-Level Proportion of Women and Underrepresented Groups in Medicine (URM) Medical School Faculty by Faculty Rank, 1990 and 2019 Junior faculty were defined as assistant professor or instructor-level faculty. Because of the high number of institutions with no women or URM chairs, some plot points are not displayed.

The greatest increases in the number of women and URM faculty were observed within junior faculty (eTable 3 in Supplement 1). For all faculty ranks among women and URM, there was an increasing proportion (vertical shift on the swarm plot) and widening distribution (vertical spread on the swarm plot) among institutions (Figure 4). Most institutions with no women or URM chairs in 1990 shifted to include women and URM chairs in 2019 (Figure 4).

## Discussion

In this retrospective cross-sectional study, we describe overall increases in women and URM faculty from 1990 to 2019. When compared with matched county demographics, URM representation increased from a median RQ of 0.16 to 0.24; however, this change was not statistically significant (*P* = .052). The trend was diminished by the growing Hispanic population in US communities compared with changes in faculty. Moreover, there was no change in RQ for White faculty, who remain highly represented. Asian faculty were highly represented compared with local demographic representation, and their presence continued to increase. We found considerable institutional variability in representation for women and URM as well as variable trends over time. Finally, we found that county and national-based RQs differed considerably, and comparisons with regional populations may provide a distinct context with respect to representational trends.

This study is among the few to evaluate trends of women and URM faculty in the context of comparator populations and the first, to our knowledge, to assess these changes on an institutional level, thereby providing insight into variability across US medical schools. Our results echo prior studies that evaluated these trends in aggregate^12,13,20^ or on a departmental level,^15,16^ in comparison with national demographics, confirming major improvements in representation of women, but lack of substantive progress for URM^15,20^ and declining trend in representation of the Hispanic population.^15^ However, we also show the presence of considerable variation in the growth and representation of URM across institutions, which implies the presence of positive factors and policies that resulted in successful recruitment and retention of underrepresented faculty. We found that high representation in earlier years was associated with higher representation in 2019 for both women and URM, which may suggest the presence of deeply rooted directives and a culture dedicated to promoting diversity.

Seemingly paradoxically, we also found that schools with higher *US News & World Report* research rankings and larger faculty rosters tended to have lower RQs. This likely represents the impact of the variability in county diversity on the RQ metric, as schools with better rankings and larger faculty rosters generally reside in highly diverse cities. It cannot be determined from our study how critical these factors are for the recruitment and retention of URM faculty; however, our findings may suggest that solely having more resources (eg, research funding, faculty mentors) is not sufficient to bolster URM faculty at rates commensurate to local demographic changes.

These findings lay a foundation for future studies and initiatives to identify and apply insights that will create transparency in faculty representation and bolster efforts to improve diversity in medicine. A potential next step may involve analysis of individual-level data to identify the recruitment and retention patterns of URM trainees across institutions. Qualitative methods may then ascertain common themes among successful and struggling institutions. Improvement of URM representation within medical school faculty is critical because of faculty connections to culture. Additionally, URM faculty are better equipped to understand lived experience with racism, trust in the health system (or lack thereof), implicit or explicit bias by physicians who are not underrepresented, and structural racism.

The AAMC defines URM as “racial and ethnic populations that are underrepresented in the medical profession relative to their numbers in the general population.”^21^ However, there is no standard method to make this comparison, and different comparator groups may provide unique insights. In our study, we assessed faculty representation in the context of county demographics and found this RQ derivation to vary considerably from a nationally based metric. This framework not only aligns with the AAMC’s objective to shift focus from a national to a regional perspective^21^ but also possesses several advantages. First, many medical institutions are located in more diverse urban areas, which would be expected to be more diverse on average when compared with national statistics. Second, certain counties may have demographic patterns that diverge from national averages, which may engender unique clinical and research missions. Such missions may be enhanced by the perspectives and experiences of faculty who have personal connections into racial, ethnic, and cultural nuances that influence health access and outcomes,^6^ thereby driving a distinct need for one institution compared with another situated in a different environment. Third, local demographics and related characteristics may be mediating factors in recruitment and retention of underrepresented faculty who may be motivated to seek and maintain employment in institutions surrounded by communities with which they can identify.^17,22,23^ Certain institutions may experience challenges in obtaining and keeping underrepresented faculty due to these reasons, and tailored solutions may be required to bolster URM employment within these schools.

However, despite these advantages, county-level data may be misleading in instances of large population shifts, which may be due to natural influx and efflux of certain populations or boundary changes. Additionally, these data do not capture the complete catchment of institutions, which may extend to areas beyond the immediate county. In some circumstances, county demographics may be much less diverse than what is seen nationally, setting a standard of racial and ethnic representation that could be misaligned with these institutions’ broader social mission to improve health equity. Institutions define their missions differently and may prioritize specific social responsibilities (ie, responsibilities to the immediate region, nationally or even globally). Hence, a generalized benchmark (ie, national) would not adequately capture these additional aspects that might define an individual institution’s representational goal. A county-based comparison may be a step further toward individualizing these metrics, but there are undoubtedly more nuanced means to measure representational goals for each school. This might include a uniquely constructed benchmark, or combination, that is weighted to reflect an institution’s objectives.

Finally, because a school’s representational goal would be expected to reflect their underlying mission, there should be transparency regarding this goal and the progress made. Some institutions already offer internal data regarding faculty composition^24,25^: a kind of scorecard, report, or audit regarding diversity, equity, and inclusion. These metrics provide insights for the region and would be enhanced by the presence of a mission-driven benchmark, which may promote community interest and engagement in this critical issue. Openness and transparency may be important catalysts to solving inequities within US medical schools.

### Limitations

Our study had several limitations in addition to those stated above. First, our analysis excludes 37 colleges of osteopathic medicine, which comprise a substantial portion of the population of interest and are responsible for training many physicians and researchers. Exclusion was based on the limited data available. Second, our data do not allow us to distinguish between basic science and clinical faculty. Basic science departments have poorer representation of women and URM relative to clinical departments,^13^ and therefore, their inclusion likely led to our analysis underestimating changes in diversity in the academic clinician workforce. However, diversity within basic science faculty remains as important an issue to drive research and health care innovation in a direction that is equitably applicable and relevant to all, and not just select populations. Recruitment of underrepresented populations in clinical trials remains problematic,^26^ and studies suggest that representation among researchers may improve willingness to participate.^27^ Finally, US Census decennial data (1990, 2000, and 2010 in our study) have historically undercounted members of certain minority groups, namely Black and Hispanic populations,^28^ which may have led to an overestimation of progress for URM groups in our study. We otherwise did not match populations by age due to lack of such data for faculty, which could have had a contrasting effect for Hispanic representation. The inclusion of the US Hispanic youth (ie, younger than 18 years), which comprises one-third of this population,^29^ results in underestimation of their RQ, as these individuals have not yet chosen careers. However, a prior study matching for age did not differ in their conclusions regarding the downward trend of Hispanic representation.^15^

## Conclusions

The findings of this cross-sectional study suggest that little progress has been made in URM representation among medical school faculty when contextualized with matched regional demographics. Though modest increases in representation were noted for Black faculty, Hispanic faculty experienced declining representation. We confirmed that institutional variability is high. Representation of women has improved, but most institutions still fall below the level of equality. Only a small number of institutions experienced an increase in URM representation, and most institutions have an RQ of less than 0.50 in 2019. Additional studies are needed to ascertain differences that allowed certain institutions to effectively increase their ranks. We also found that county-level population comparisons function differently than national comparisons in assessing medical faculty representation for women and URM. County-level benchmarks may be helpful in assessing representation in the context of regional demographics.

## Notes

Supplement 1. **eMethods.** Data Sources, Institutional Characteristics, and Inclusion and Exclusion Criteria **eFigure 1.** Study Selection Flow Diagram **eFigure 2.** Trends in Number of Institutions Included in the Analysis, 1990 to 2019 **eFigure 3.** Institutional Ranking by Change in Representation Quotient for Women and URM Medical School Faculty, 1990 to 2019 **eFigure 4.** Representation of URM Individuals Within County vs Associated Institutions, Ranked by County in 1990 **eTable 1.** Medical School Faculty Representation Quotient for Women and URM by Institution Characteristics, 2019 **eTable 2.** Medical School Faculty Representation Quotient for Women and URM by Institution Faculty and Student Composition, 2019 **eTable 3.** Institutional Proportion of Medical School Faculty for Women and Race and Ethnicity Subgroups by Faculty Rank, 1990 to 2019
eReferences

Supplement 2.
Data Sharing Statement
