# Supplementary material for: Institutional Variability in Representation of Women and Racial and Ethnic Minority Groups Among Medical School Faculty
Source: JAMA Netw Open. 2022 Dec 20;5(12):e2247640. doi: 10.1001/jamanetworkopen.2022.47640 (PMC9857368; doi:10.1001/jamanetworkopen.2022.47640)
Supplement: Supplement 1. — eMethods. Data Sources, Institutional Characteristics, and Inclusion and Exclusion Criteria eFigure 1. Study Selection Flow Diagram eFigure 2. Trends in Number of Institutions Included in the Analysis, 1990 to 2019 eFigure 3. Institutional Ranking by Change in Representation Quotient for Women and URM Medical School Faculty, 1990 to 2019 eFigure 4. Representation of URM Individuals Within County vs Associated Institutions, Ranked by County in 1990 eTable 1. Medical School Faculty Representation Quotient for Women and URM by Institution Characteristics, 2019 eTable 2. Medical School Faculty Representation Quotient for Women and URM by Institution Faculty and Student Composition, 2019 eTable 3. Institutional Proportion of Medical School Faculty for Women and Race and Ethnicity Subgroups by Faculty Rank, 1990 to 2019 eReferences [file jamanetwopen-e2247640-s001.pdf]

Supplementary Online Content

Yoo A, Auinger P, Tolbert J, Paul D, Lyness JM, George BP. Institutional variability in representation of women and racial and ethnic minority groups among medical school faculty. *JAMA Netw Open*. 2022;5(12):e2247640.
doi:10.1001/jamanetworkopen.2022.47640

eMethods. Data Sources, Institutional Characteristics, and Inclusion and Exclusion Criteria
eFigure 1. Study Selection Flow Diagram
eFigure 2. Trends in Number of Institutions Included in the Analysis, 1990 to 2019
eFigure 3. Institution Rank by Change in Representation Quotient for Women and URM Medical School Faculty, 1990 to 2019
eFigure 4. Representation of URM Individuals Within County vs Associated Institutions, Ranked by County in 1990
eTable 1. Medical School Faculty Representation Quotient for Women and URM by Institutional Characteristics, 2019
eTable 2. Medical School Faculty Representation Quotient for Women and URM by Institution Faculty and Student Composition, 2019
eTable 3. Institutional Proportion of Medical School Faculty for Women and Race and Ethnicity Subgroups by Faculty Rank, 1990 to 2019
eReferences

This supplementary material has been provided by the authors to give readers additional information about their work.

© 2022 Yoo A et al. *JAMA Network Open*.

eMethods. Data Sources, Institutional Characteristics, and Inclusion and Exclusion Criteria

Data Sources

AAMC FAMOUS data included total full-time medical school faculty aggregated at the institution-level, extracted by two separate queries: (1) by sex and faculty rank, and (2) by race and ethnicity and faculty rank for each year, 1990-2019. The datasets by individual institution were maintained separately for sex (male, female) and race and ethnicity (i.e., American Indian or Native Alaskan [AIAN], Asian, Black, Hispanic, Native Hawaiian or Oceanic Pacific Islander [NHOPI], White). Faculty reporting as "multiple race Hispanic" were grouped with those identifying as Hispanic. Therefore, faculty members of any single race who also identified as Hispanic ethnicity, were grouped with those identifying as Hispanic. We defined URM as AIAN, Black, Hispanic, or NHOPI, consistent with prior studies.[1–4] Faculty rank was available as Department Chair, Professor, Associate, Assistant, and Instructor ranks. For our study, Assistant and Instructor ranks were combined to "Junior Faculty".

Institution ZIP codes were identified using the AAMC member website and matched to the corresponding county for each medical school using a government-sourced crosswalk.[5] County demographics were extracted from the US Census Bureau Population and Housing Unit Estimate: Intercensal Datasets from 1990 to 2019 except for years 1990, 2000, 2010 in which decennial census count data were available. Prior to 2000, the US Census Bureau grouped Asian and Native Hawaiian or Oceanic Pacific Islanders (NHOPI) in the same category, resulting in population counts and estimates only being available for NHOPI individuals from 2000-2019.

Institutional Characteristics

We collected data using publicly available sources[6] to identify public/private ownership, campus setting (i.e., urban/suburban/rural), US Region (Northeast, Midwest, South, West), Historically

© 2022 Yoo A et al. *JAMA Network Open*.

Black College and University (HBCU) status, year of establishment, *US News and World Report* research/primary care rankings (divided into quartiles where the bottom quartile represents unranked institutions). We grouped institutions into quartiles based on faculty size, proportion of women and URM in professor rank in the earliest year of data for each institution, proportion of women and URM medical students in 2019, and the RQ for women and URM in the earliest year. We additionally examined the presence of women and URM department chairs in the earliest year of data for each institution.

Inclusion/Exclusion

Allopathic US Medical Schools with three or more years of data in the AAMC FAMOUS database were included in the analysis. There were seven institutions from 1990 to 2019 (n=1,257 faculty entries) excluded due to insufficient consecutive years of data (<3 years) to establish trends. There were four institutions in which the first year of data entry demonstrated <20 faculty leading to outlier calculations, and therefore, these years were excluded for these institutions. We also excluded medical schools located in Puerto Rico (four schools from 1990-2019, n=20,944 faculty entries). There were 144 institutions and 3,729,888 faculty entries included over the timeframe of the study (n=121 institutions with complete data for the 30-year timeframe) [**eFigure 1**]. After matching to county-level population data and institutional variables, institutions were deidentified to maintain anonymity. Faculty identified as multiple race non-Hispanic (1.7% of total), other or unknown race and ethnicity (3.8% of total), or unknown sex (1.4% of total) were not analyzed separately in this study but are included in the denominator of total faculty for the purpose of calculations

© 2022 Yoo A et al. *JAMA Network Open*.

eFigure 1. Study Selection Flow Diagram

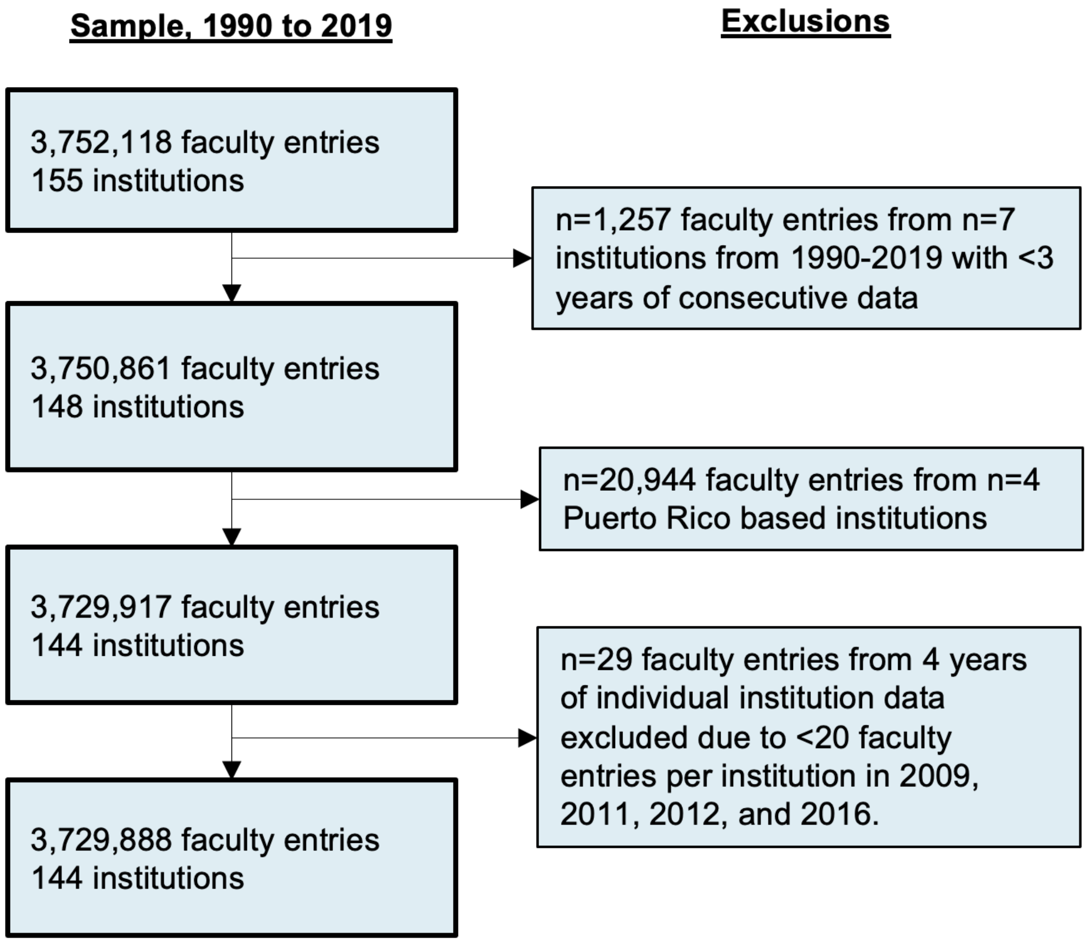

Faculty entries are individual counts entered into the Association of American Medical Colleges (AAMC) Faculty Administrative Management Online User System (FAMOUS) from 1990 to 2019. A single individual may be included up to 30 times (once per year) within the dataset. Data were obtained aggregated on the institution-level per year.

© 2022 Yoo A et al. *JAMA Network Open*.

eFigure 2. Trends in Number of Institutions Included in the Analysis, 1990 to 2019

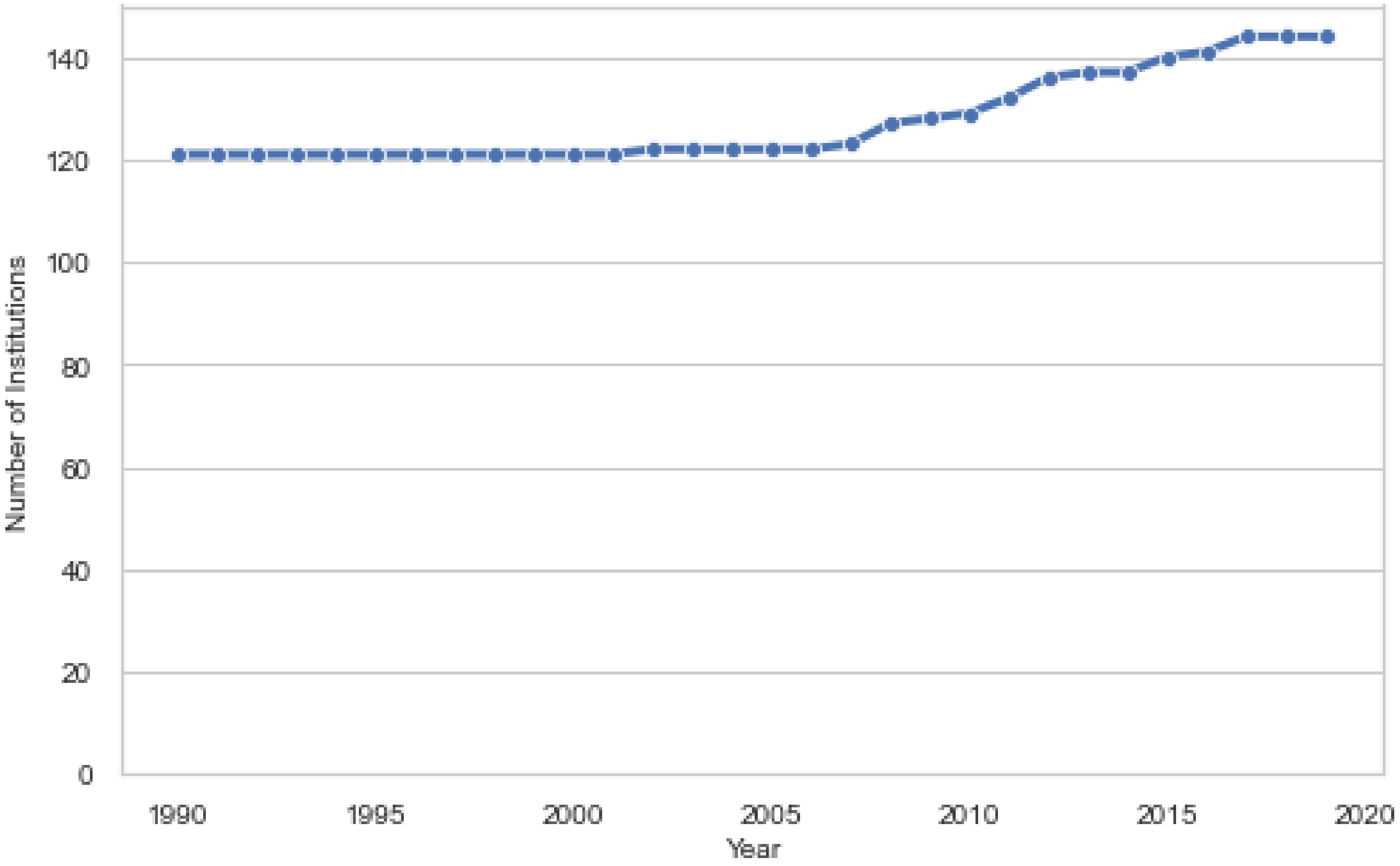

After study selection methods, there were 121 Association of American Medical Colleges (AAMC) member institutions within the Faculty Administrative Management Online User System (FAMOUS) dataset in 1990 and 144 AAMC member institutions in 2019. This graph demonstrates the years new institutions entered the dataset.

© 2022 Yoo A et al. *JAMA Network Open*.

eFigure 3. Institution Rank by Change in Representation Quotient for Women and URM Medical School Faculty, 1990 to 2019

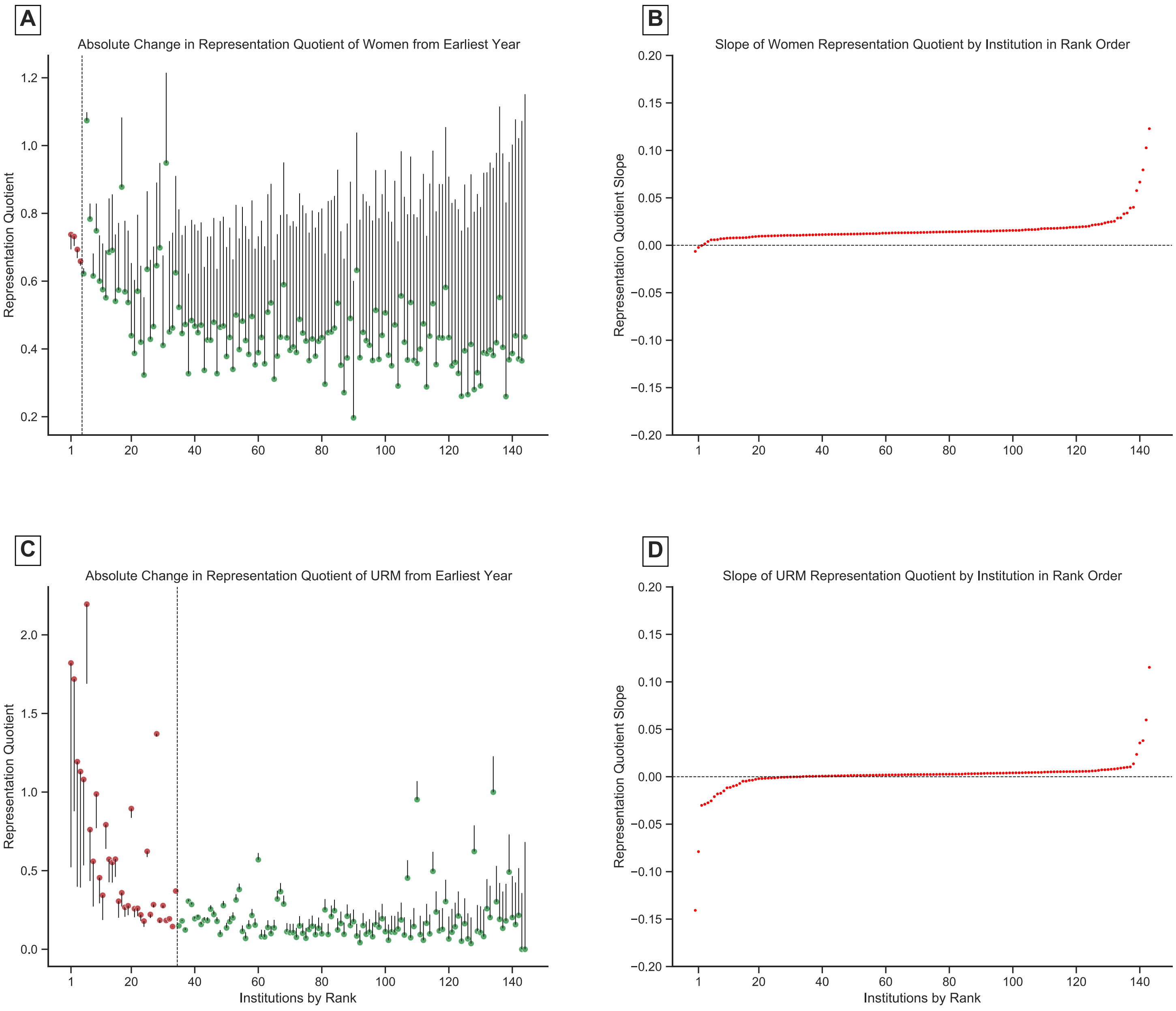

Abbreviation: URM = Underrepresented in Medicine. (A and C) Absolute change in representation quotient was calculated as the difference between 1990 and 2019 and displayed in institution rank order of vector magnitude and direction. The vertical dotted line separate institutions with negative (left) vs. positive (right) change from 1990 to 2019. (B and D) Slope estimate is the change per year as calculated by linear mixed-effects models with a maximum likelihood approach and an autoregressive correlation AR(1) structure with year (i.e., 1990-2019) as the dependent variable and institution as the repeated measure. Institutions with a value and 95% confidence interval above zero indicates positive change (n=X for women, n=X for URM), confidence interval overlapping zero indicates lack of significance or no change (n=Y for women, n=Y for URM, and below zero indicate negative change (n=Z for women and n=Z for URM

© 2022 Yoo A et al. *JAMA Network Open*.

eFigure 4. Representation of URM Individuals Within County vs Associated Institutions, Ranked by County in 1990

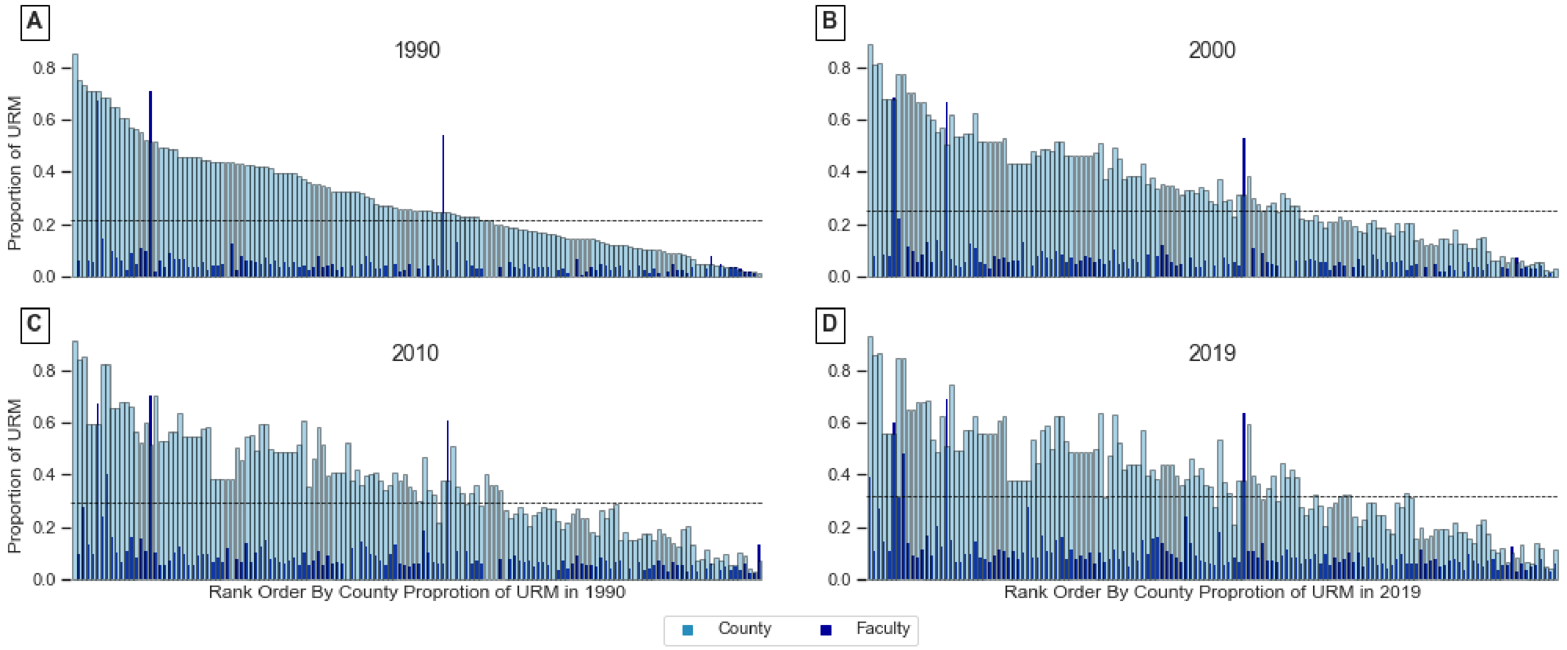

Abbreviation: URM = Underrepresented in Medicine. (A-D) Proportion of URM in county (represented by light blue) with proportion of URM among medical school faculty (represented by dark blue) presented as an overlapped bar chart. Institutions are ordered in descending order of URM representation within the associated county in 1990. This order is preserved for subplots B-D. The horizontal dotted reference line provides a national average of URM representation by decade, calculated from the decennial US census for years 1990, 2000, and 2010 and the intercensal census for year 2019.

© 2022 Yoo A et al. *JAMA Network Open*.

eTable 1. Medical School Faculty Representation Quotient for Women and URM by Institutional Characteristics, 2019

Institution Characteristics	Institutions No. (%)	Faculty Entries No. (%)	RQ Women median (IQR)	*P* value	RQ URM median (IQR)	*P* value
**Total**	144 (100)	184,577 (100)	0.80 (0.74-0.89)		0.24 (0.19-0.37)	
**School Ownership**						
Public	88 (61)	92,295 (50)	0.79 (0.73-0.89)	0.43	0.28 (0.21-0.42)	<0.001
Private	56 (39)	92,282 (50)	0.81 (0.75-0.89)		0.20 (0.16-0.27)	
**Campus Setting**						
Urban	106 (74)	144,758 (78)	0.82 (0.75-0.90)	0.04	0.21 (0.18-0.32)	<0.001
Suburban	31 (22)	36,962 (20)	0.77 (0.70-0.82)		0.29 (0.21-0.42)	
Rural	7 (5)	2,857 (2)	0.76 (0.73-0.79)		0.77 (0.39-0.88)	
**Region**						
Northeast	34 (24)	56,944 (31)	0.82 (0.76-0.86)	0.005	0.25 (0.20-0.35)	0.64
Midwest	35 (24)	38,018 (21)	0.78 (0.72-0.84)		0.29 (0.18-0.46)	
South	55 (38)	59,661 (32)	0.79 (0.71-0.86)		0.23 (0.19-0.39)	
West	20 (14)	29,954 (16)	0.88 (0.81-0.98)		0.21 (0.20-0.28)	
**HBCU**						
Yes	3 (2)	583 (0.3)	0.84 (0.77-1.04)	0.37	1.35 (1.07-1.69)	0.003
No	141 (98)	183,994 (100)	0.80 (0.74-0.89)		0.24 (0.19-0.37)	
**Year Established**						
1765-1859	32 (22)	53,136 (29)	0.78 (0.73-0.87)	0.98	0.21 (0.17-0.26)	0.004
1860-1909	38 (26)	58,094 (31)	0.78 (0.75-0.89)		0.21 (0.18-0.42)	
1910-1959	17 (12)	28,574 (15)	0.80 (0.77-0.84)		0.20 (0.17-0.26)	
1960-2017	57 (40)	44,773 (24)	0.82 (0.70-0.89)		0.29 (0.21-0.39)	
**Faculty Size, qrt**						
First	36 (25)	8,358 (5)	0.81 (0.71-0.91)	0.03	0.34 (0.22-0.60)	0.002
Second	36 (25)	28,141 (15)	0.78 (0.73-0.83)		0.23 (0.19-0.36)	
Third	36 (25)	45,161 (24)	0.77 (0.73-0.86)		0.22 (0.19-0.33)	
Fourth	36 (25)	102,917 (56)	0.82 (0.79-0.92)		0.21 (0.17-0.28)	
**Research Rank 2021, qrt***						
First	38 (26)	93,833 (51)	0.82 (0.74-0.92)	0.11	0.21 (0.17-0.28)	0.02
Second	35 (24)	44,727 (24)	0.80 (0.76-0.85)		0.23 (0.19-0.29)	
Third	34 (24)	21,007 (11)	0.77 (0.70-0.83)		0.27 (0.20-0.39)	
Unranked	37 (26)	25,010 (14)	0.81 (0.76-0.89)		0.32 (0.21-0.52)	
**Primary Care Rank 2021, qrt***						
First	36 (25)	77,352 (42)	0.82 (0.77-0.92)	0.02	0.23 (0.18-0.35)	0.07
Second	32 (22)	52,140 (28)	0.80 (0.74-0.90)		0.21 (0.17-0.27)	
Third	33 (23)	26,182 (14)	0.75 (0.69-0.83)		0.26 (0.19-0.34)	
Unranked	43 (30)	28,903 (16)	0.81 (0.75-0.89)		0.30 (0.20-0.43)	

© 2022 Yoo A et al. *JAMA Network Open*.

Institution Characteristics	Institutions No. (%)	Faculty Entries No. (%)	RQ Women median (IQR)	*P* value	RQ URM median (IQR)	*P* value
**County Proportion URM, qrt**						
First	36 (25)	37,828 (20)	0.77 (0.72-0.89)	0.79	0.43 (0.37-0.55	<0.001
Second	36 (25)	51,700 (28)	0.80 (0.73-0.86)		0.21 (0.20-0.28)	
Third	36 (25)	48,418 (26)	0.82 (0.76-0.89)		0.21 (0.17-0.28	
Fourth	36 (25)	46.631 (25)	0.80 (0.75-0.86)		0.19 (0.16-0.25)	

Abbreviations: RQ = Representation Quotient, URM = Underrepresented in Medicine, IQR = Interquartile Range, HBCU = Historically Black Colleges and Universities, qrt = quartile. URM includes American Indian or Alaskan Natives (AIAN), Black, Hispanic, Native Hawaiian or Oceanic Pacific Islander (NHOPI). Comparisons of RQ across categorical variables were made using Wilcoxon Rank Sum or Kruskal-Wallis test where appropriate. A p-value of less than or equal to .05 was considered significant. Higher quartiles indicate higher faculty size and proportion of URM within the county (e.g., 4th quartile institutions would be expected to have the largest faculty rosters). Quartiles for research and primary care rank are ordered (e.g., first quartile research rank indicates the top institution)

© 2022 Yoo A et al. *JAMA Network Open*.

eTable 2. Medical School Faculty Representation Quotient for Women and URM by Institution Faculty and Student Composition, 2019

Faculty and Student Composition	Institutions No. (%)	Faculty Entries No. (%)	RQ Female median (IQR)	*P* value	RQ URM median (IQR)	*P* value
**Women Department Chair Present in Earliest Year**						
Yes	84 (58)	123,186 (67)	0.80 (0.74-0.89)	0.40	0.24 (0.19-0.37)	0.88
No	60 (42)	61,391 (33)	0.80 (0.72-0.86)		0.24 (0.18-0.37)	
**URM Department Chair Present in Earliest Year**						
Yes	52 (36)	66,638 (36)	0.80 (0.73-0.86)	0.78	0.24 (0.19-0.37)	0.88
No	92 (64)	117,939 (64)	0.80 (0.74-0.89)		0.24 (0.18-0.37)	
**Proportion Women Professor in Earliest Year, qrt**						
First	36 (25)	48,361 (26)	0.79 (0.71-0.85)	0.20	0.24 (0.18-0.36)	0.19
Second	36 (25)	47,916 (26)	0.81 (0.74-0.87)		0.22 (0.18-0.29)	
Third	36 (25)	53,662 (29)	0.82 (0.76-0.92)		0.21 (0.19-0.37)	
Fourth	36 (25)	34,638 (19)	0.80 (0.73-0.90)		0.31 (0.21-0.42)	
**Proportion URM Professor in Earliest Year, qrt**						
First	36 (25)	49,302 (27)	0.79 (0.71-0.89)	0.73	0.21 (0.19-0.32)	0.08
Second	36 (25)	56,526 (31)	0.80 (0.75-0.89)		0.25 (0.18-0.37)	
Third	37 (26)	51,004 (28)	0.81 (0.77-0.86)		0.22 (0.18-0.29)	
Fourth	35 (24)	27,745 (15)	0.80 (0.71-0.89)		0.31 (0.20-0.52)	
**Proportion Women Students in 2019, qrt**						
First	37 (26)	41,363 (22)	0.78 (0.72-0.84)	0.10	0.21 (0.19-0.30)	0.005
Second	35 (24)	46,457 (25)	0.80 (0.73-0.87)		0.26 (0.18-0.43)	
Third	36 (25)	54,900 (30)	0.80 (0.74-0.87)		0.21 (0.18-0.29)	
Fourth	36 (25)	41,857 (23)	0.83 (0.77-0.96)		0.30 (0.23-0.44)	
**Proportion URM Students in 2019, qrt**						
First	36 (25)	28,986 (16)	0.77 (0.73-0.87)	0.43	0.24 (0.18-0.42)	0.23
Second	36 (25)	51,226 (28)	0.80 (0.74-0.92)		0.23 (0.20-0.29)	
Third	36 (25)	69,264 (38)	0.81 (0.73-0.86)		0.21 (0.18-0.29)	
Fourth	36 (25)	35,101 (19)	0.82 (0.77-0.89)		0.29 (0.20-0.44)	
**Women RQ in Earliest Year, qrt**						
First	36 (25)	41,479 (22)	0.77 (0.69-0.81)	0.01	0.24 (0.19-0.37)	0.12
Second	36 (25)	51,405 (28)	0.80 (0.73-0.89)		0.21 (0.17-0.29)	
Third	36 (25)	59,299 (32)	0.82 (0.77-0.86)		0.23 (0.19-0.41)	
Fourth	36 (25)	32,394 (18)	0.85 (0.74-0.96)		0.29 (0.20-0.40)	
**URM RQ in Earliest Year, qrt**						
First	36 (25)	62,118 (34)	0.79 (0.75-0.91)	0.76	0.17 (0.16-0.21)	<0.001
Second	36 (25)	51,187 (28)	0.81 (0.74-0.86)		0.21 (0.18-0.25)	
Third	36 (25)	44,562 (24)	0.81 (0.77-0.89)		0.26 (0.21-0.32)	
Fourth	36 (25)	26,710 (14)	0.77 (0.71-0.89)		0.45 (0.37-0.68)	

Abbreviations: RQ = Representation Quotient, URM = Underrepresented in Medicine, IQR = Interquartile Range, qrt = quartile. URM includes American Indian or Alaskan Natives (AIAN), Black, Hispanic, Native Hawaiian or Oceanic Pacific Islander (NHOPI). Comparisons of RQ across categorical variables were made using Wilcoxon Rank Sum or Kruskal-Wallis test where appropriate. A p-value of less than or equal to .05 was considered significant.

© 2022 Yoo A et al. *JAMA Network Open*.

eTable 3. Institutional Proportion of Medical School Faculty for Women and Race and Ethnicity Subgroups by Faculty Rank, 1990 to 2019

	Proportion Junior Faculty, median (IQR)		Slope Estimate	*P* value	Proportion Associate Professor, median (IQR)		Slope Estimate	*P* value
	1990	2019			1990	2019		
Women	0.25 (0.22-0.27)	0.45 (0.41-0.48)	0.007	<0.001	0.18 (0.15-0.22)	0.38 (0.35-0.43)	0.007	<0.001
Underrepresented in Medicine	0.04 (0.03-0.06)	0.09 (0.07-0.12)	0.002	<0.001	0.04 (0.02-0.05)	0.08 (0.06-0.11)	0.002	<0.001
Race and Ethnicity								
AIAN	0.00 (0.00-0.002)	0.001 (0.00-0.002)	<0.001	0.029	0.00 (0.00-0.00)	0.00 (0.00-0.003)	<0.001	<0.001
Asian	0.07 (0.06-0.10)	0.22 (0.18-0.26)	0.005	<0.001	0.07 (0.05-0.10)	0.19 (0.14-0.24)	0.004	<0.001
Black	0.02 (0.01-0.03)	0.04 (0.03-0.06)	0.001	<0.001	0.01 (0.005-0.02)	0.03 (0.02-0.05)	0.001	<0.001
Hispanic	0.02 (0.01-0.03)	0.05 (0.04-0.06)	0.001	<0.001	0.02 (0.01-0.03)	0.04 (0.03-0.06)	0.001	<0.001
NHOPI	0.00 (0.00-0.00)	0.00 (0.00-0.001)	<0.001	0.015	No faculty	0.00 (0.00-0.00)	<0.001	<0.001
White	0.85 (0.80-0.88)	0.61 (0.54-0.68)	-0.007	<0.001	0.86 (0.81-0.89)	0.67 (0.59-0.73)	-0.006	<0.001

	Proportion Full Professor, median (IQR)		Slope Estimate	P value	Proportion Department Chair, median (IQR)		Slope Estimate	P value
	1990	2019			1990	2019		
Women	0.08 (0.06-0.10)	0.25 (0.22-0.28)	0.006	<0.001	0.04 (0.00-0.06)	0.20 (0.15-0.26)	0.006	<0.001
Underrepresented in Medicine	0.02 (0.01-0.04)	0.05 (0.04-0.08)	0.001	<0.001	0.00 (0.00-0.05)	0.06 (0.00-0.10)	0.001	<0.001
Race and Ethnicity								
AIAN	0.00 (0.00-0.00)	0.00 (0.00-0.00)	<0.001	0.14	0.00 (0.00-0.00)	0.00 (0.00-0.00)	<0.001	0.85
Asian	0.05 (0.03-0.07)	0.13 (0.10-0.17)	0.003	<0.001	0.00 (0.00-0.04)	0.09 (0.04-0.13)	0.003	<0.001
Black	0.005 (0.00-0.01)	0.02 (0.01-0.03)	<0.001	<0.001	0.00 (0.00-0.00)	0.00 (0.00-0.05)	0.001	<0.001
Hispanic	0.02 (0.005-0.03)	0.03 (0.02-0.05)	0.001	<0.001	0.00 (0.00-0.00)	0.02 (0.00-0.06)	0.001	<0.001
NHOPI	No faculty	0.00 (0.00-0.00)	<0.001	0.71	No faculty	0.00 (0.00-0.00)	<0.001	0.18
White	0.90 (0.86-0.93)	0.76 (0.71-0.81)	-0.004	<0.001	0.94 (0.90-1.00)	0.82 (0.73-0.89)	-0.005	<0.001

Abbreviations: IQR = Interquartile Range, AIAN = American Indian or Alaskan Native, NHOPI = Native Hawaiian or Oceanic Pacific Islander. Underrepresented in Medicine includes AIAN, Black, Hispanic, NHOPI. If the median value or 25th to 75th percentile was zero, then a zero value is shown. There were no faculty identifying with NHOPI in Associate, Professor, or Department Chair rank for any institutions in 1990 and therefore "No faculty" is displayed. Junior faculty are those identified as Assistant Professor or Instructor. Slope estimate is the change per year as calculated by linear mixed-effects models with a maximum likelihood approach and an autoregressive correlation AR(1) structure with year (i.e., 1990-2019) as the dependent variable and institution as the repeated measure. Slopes were compared to zero with Student T-test to determine significance. A p-value of less than or equal to 0.05 was considered significant.

© 2022 Yoo A et al. *JAMA Network Open*.

eReferences

1. Xierali IM, Nivet MA, Wilson MR. Current and Future Status of Diversity in Ophthalmologist Workforce. *JAMA Ophthalmol*. 2016;134(9):1016. doi:10.1001/jamaophthalmol.2016.2257
2. Xierali IM, Nivet MA, Rayburn WF. Full-Time Faculty in Clinical and Basic Science Departments by Sex and Underrepresented in Medicine Status: A 40-Year Review. *Academic Medicine*. 2021;96(4):568-575. doi:10.1097/ACM.0000000000003925
3. Yoo A, George BP, Auinger P, Strawderman E, Paul DA. Representation of Women and Underrepresented Groups in US Academic Medicine by Specialty. *JAMA Netw Open*. 2021;4(8):e2123512. doi:10.1001/jamanetworkopen.2021.23512
4. Ogunwole SM, Dill M, Jones K, Golden SH. Trends in Internal Medicine Faculty by Sex and Race/Ethnicity, 1980-2018. *JAMA Netw Open*. 2020;3(9):e2015205. doi:10.1001/jamanetworkopen.2020.15205
5. HUD USPS ZIP CODE CROSSWALK FILES. Accessed June 17, 2021. https://www.huduser.gov/portal/datasets/usps_crosswalk.html
6. Morse R, Vega-Rodriguez J, Castonguay A, Brooks E, Hines K. Methodology: 2022 Best Medical Schools Rankings. Find out how U.S. News ranks medical schools. Published March 29, 2021. Accessed March 27, 2022. https://www.usnews.com/education/best-graduate-schools/articles/medical-schools-methodology

© 2022 Yoo A et al. *JAMA Network Open*.
