# Supplementary material for: Institutional Variability in Representation of Women and Racial and Ethnic Minority Groups Among Medical School Faculty
Source: JAMA Netw Open. 2022 Dec 20;5(12):e2247640. doi: 10.1001/jamanetworkopen.2022.47640 (PMC9857368; doi:10.1001/jamanetworkopen.2022.47640)
Supplement: Supplement 2. — Data Sharing Statement [file jamanetwopen-e2247640-s002.pdf]

Data Sharing Statement

Yoo. Institutional Variability in Representation of Women and Racial and Ethnic Minority Groups Among Medical School Faculty. *JAMA Netw Open*. Published December 20, 2022. doi:10.1001/jamanetworkopen.2022.47640

Data
Data available: No
